# In Vitro Anthelmintic Effect of Mexican Plant Extracts and Partitions Against *Trichinella spiralis* and *Strongyloides venezuelensis*

**DOI:** 10.3390/plants13243484

**Published:** 2024-12-12

**Authors:** Nancy E. Rodríguez-Garza, Ricardo Gomez-Flores, Ramiro Quintanilla-Licea, Joel H. Elizondo-Luévano, César I. Romo-Sáenz, Miguel Marín, Javier Sánchez-Montejo, Antonio Muro, Rafael Peláez, Julio López-Abán

**Affiliations:** 1 Departamento de Microbiología e Inmunología, Facultad de Ciencias Biológicas, Universidad Autónoma de Nuevo León, San Nicolás de los Garza 66455, Nuevo León, Mexico; nancy.rodriguezgrz@uanl.edu.mx (N.E.R.-G.); ricardo.gomezfl@uanl.edu.mx (R.G.-F.); cesar.romosnz@uanl.edu.mx (C.I.R.-S.); 2 Grupo de Enfermedades Infecciosas y Tropicales (e-INTRO), Instituto de Investigación Biomédica de Salamanca—Centro de Investigación de Enfermedades Tropicales de la Universidad de Salamanca (IBSAL-CIETUS), Facultad de Farmacia, Universidad de Salamanca, 37007 Salamanca, Spain; joel.elizondolv@uanl.edu.mx (J.H.E.-L.); mmarin@usal.es (M.M.); s.montejo@usal.es (J.S.-M.); ama@usal.es (A.M.); 3 Departamento de Química, Facultad de Ciencias Biológicas, Universidad Autónoma de Nuevo León, San Nicolás de los Garza 66455, Nuevo León, Mexico; ramiro.quintanillalc@uanl.edu.mx; 4 Facultad de Medicina y Ciencias Biomédicas, Universidad Autónoma de Chihuahua, Chihuahua 31109, Chihuahua, Mexico; 5 Laboratorio de Química Orgánica y Farmacéutica, Departamento de Ciencias Farmacéuticas, Facultad de Farmacia, Universidad de Salamanca, 37007 Salamanca, Spain

**Keywords:** nematocidal activity, parasitic activity, medicinal plants, methanol extract, *Trichinella spiralis*, *Strongyloides venezuelensis*

## Abstract

Parasitic diseases represent a significant global public health concern. Two clinically important parasites of high prevalence rates are *Trichinella spiralis* and *Strongyloides stercoralis*. However, the limitations of currently used nematocidal drugs highlight the urgent need for novel treatment approaches. The present study investigated the in vitro nematocidal activity of methanol extracts from *Amphipterygium adstringens*, *Artemisia ludoviciana*, *Cymbopogon citratus*, *Heterotheca inuloides*, *Jatropha dioica*, *Justicia spicigera*, *Larrea tridentata*, *Mimosa tenuiflora*, *Psacalium decompositum*, *Ruta chalepensis*, *Semialarium mexicanum*, and *Smilax aspera* against *T. spiralis* L1 and *S. venezuelensis* L3 (model for *S. stercoralis*). Most of the plants showed antiparasitic activity, but *R. chalepensis* crude methanol extract showed the most potent nematocidal activity against both parasites, with a mean lethal concentration (LC_50_) of 28.2 µg/mL and a selectivity index (SI) of 22.4 for *T. spiralis* and an LC_50_ of 244.8 µg/mL and SI of 2.58 for *S. venezuelensis*. This extract was further separated into *n*-hexane, chloroform, and methanol partitions by continuous Soxhlet extractions. The *n*-hexane partition demonstrated the strongest activity against both parasites, with an LC_50_ of 147.6 µg/mL and an SI of 7.77 against *T. spiralis* and an LC_50_ of 39.2 µg/mL and an SI of 3.77 against *S. venezuelensis*. LC-MS/MS analysis identified coumarins as the main chemical class (53%), and chalepin represented this partition’s most abundant compound (29.9%). Overall, this study confirmed the antiparasitic potential of medicinal plants commonly used in Mexico. In addition, it highlights the possibility of obtaining bioactive compounds from plants like *R. chalepensis,* or the other plants evaluated in this study, as novel treatments against parasitic diseases.

## 1. Introduction

Parasitic nematodes are a major public health concern worldwide, affecting around two billion people, primarily in developing countries [1]. Two clinically important parasites of high prevalence rates are *Trichinella spiralis* and *Strongyloides stercoralis*. *T. spiralis* is a foodborne zoonotic nematode that has infected around 11 million humans globally [2]. Humans become infected by consuming raw or undercooked pork containing *T. spiralis* larvae [3], leading to trichinellosis, which has two clinical manifestations, gastrointestinal and systemic [4]. During the gastrointestinal phase, patients show abdominal pain, diarrhea, nausea, and vomiting. In the systemic phase, larvae enter the lymphatic system and the bloodstream, reaching skeletal muscles, myocardium, and brain tissues, causing fevers, myositis, myalgias, periorbital edema, myocarditis, and encephalitis [5]. The severity of the disease directly correlates with the number of larvae ingested, with factors such as the host’s age, sex, nutrition, hormonal condition, immunity, and tissue invasion [6]. The infection may be fatal in people infected with many larvae [5]. On the other side, *S. stercoralis* is a soil-transmitted helminth that infects about 370 million people worldwide, particularly in tropical and subtropical countries, where 10% to 40% of the population is affected [7]. It is transmitted through the penetration of infective larvae into human skin upon contact with contaminated soil, or more rarely, through ingestion of contaminated food and water [8]. Strongyloidiasis has variable clinical manifestations. In immunocompetent individuals, the disease ranges from asymptomatic to mild gastrointestinal, pulmonary, and cutaneous symptoms. In contrast, immunocompromised patients could develop severe clinical forms, including hyperinfection syndrome and disseminated strongyloidiasis, which may be fatal due to the autoinfection life cycle [9,10].

To date, benzimidazole derivatives such as albendazole, mebendazole, flubendazole, and thiabendazole are the main anthelmintic drugs used to treat trichinellosis [11,12]. However, they have a limited effect against encysted larvae [13], which is potentially related to their low bioavailability [14]. Ivermectin is the treatment of choice for *S. stercoralis* infections, as it targets both adult worms and larvae. Second-line therapy is albendazole, since it is less effective than ivermectin and primarily targets adult parasites. Thiabendazole may also be used, but it is commonly replaced by ivermectin due to its gastrointestinal side effects [11]. However, the adverse effects and inconsistent cure rates of these drugs, along with the lack of information regarding their safety in pregnant and lactating women and children under the age of five years, often lead to reduced compliance and discouraged use among patients [12]. This emphasizes the need to search for new drugs against this parasite.

Around the world, numerous medicinal herbs have been used to treat different diseases [15]. Modern medicines are often derived from herbs based on traditional knowledge and practices. Approximately 25% of the major pharmaceutical compounds currently available were obtained from natural resources [16]. The anthelmintic activity of plant extracts is related to the presence of biologically active metabolites, such as tannins, flavonoids, coumarins, steroids, terpenoids, alkaloids, and saponins [17]. Examples of plant-derived antiparasitic agents currently marketed include artemisinin, originally extracted from *Artemisia annua* [18], and quinine, isolated from *Cinchona* sp., both widely used in the treatment of *Plasmodium* infections [19].

Mexico is the second country worldwide in the use of medicinal plants. As a country with a diverse range of environmental conditions, it boasts significant biological diversity, notably in its flora. Approximately 4500 species of ethnobotanical significance grow within its territory [20].

Our research group has previously evaluated the antitumoral, cytotoxic, antioxidant, hemolytic, and anti-hemolytic activity of a selected group of ethnomedicinal Mexican plants [21,22]. To further determine the ethnopharmacological effect of these plants, the aim of this study was to evaluate their in vitro nematocidal activity against *T. spiralis* and *S. venezuelensis* (model for *S. stercoralis*). These two parasites were chosen to explore a broad-spectrum nematocidal activity since one is a muscular parasite and the other gastrointestinal.

## 2. Results

### 2.1. Plant Material Identification and Plant Extract Yields

Table 1 provides the taxonomic information of the 12 plants evaluated in this study, along with their primary ethnomedical uses. Gastrointestinal disorders are often closely associated with the presence of intestinal parasites. Consequently, plants traditionally used for the treatment of gastrointestinal conditions may possess potential antiparasitic properties, warranting further investigation.

Plant extracts show different yields ranging between 11.14% and 27.37%. *S. mexicanum* showed the lowest yield (11.14%), whereas *J. spicigera* showed the highest yield (27.37%). These yields were influenced by the part of the plant used and the secondary metabolites present in each plant.

### 2.2. Nematocidal Activity Against Trichinella spiralis

We evaluated the nematocidal activity of crude methanol plant extracts against *T. spiralis* infective first-stage larvae (L1) (Table 2). The most active extracts against *T. spiralis* were *R. chalepensis* (LC_50_ = 28.2 µg/mL), *J. spicigera* (LC_50_ = 44.8 µg/mL), *J. dioica* (LC_50_ = 134.8 µg/mL), *P. decompositum* (LC_50_ = 148.5 µg/mL), *S. aspera* (LC_50_ = 188.8 µg/mL), and *L. tridentata* (LC_50_ = 450.6 µg/mL), whereas the other plant extracts did not affect *T. spiralis* (LC_50_ > 1000 µg/mL). In addition, most of the evaluated plant extracts were more toxic to *T. spiralis* than to mammalian cells (Vero), as evidenced by their IC_50_ values. Toxicity of plant extracts against Vero cells was used to determine the selectivity indexes (SIs). In this regard, Table 2 shows significant SIs of *R. chalepensis*, *P. decompositum*, *S. aspera*, *J. spicigera*, *H. inuloides*, and *L. tridentata*.

After identifying the crude extract of *R. chalepensis* in methanol with the highest SI, it was concentrated to dryness and then subjected to a continuous Soxhlet extraction process using solvents of increasing polarity (*n*-hexane, chloroform, and methanol) and evaluated the resulting partitions against *T. spiralis.* The results are presented in Table 3. The methanolic partition showed the highest yield, followed by *n*-hexane and chloroform partitions. In addition, the *n*-hexane partition showed the highest activity (LC_50_ = 19.0 µg/mL), followed by chloroform (LC_50_ = 29.3 µg/mL) and methanol (LC_50_ = 161.5 µg/mL) partitions. Furthermore, the *n*-hexane partition presented the highest SI, followed by the methanol and chloroform partitions against *T. spiralis* (Table 3).

### 2.3. Nematocidal Activity Against Strongyloides venezuelensis

We also evaluated the nematocidal activity of plant extracts against *S. venezuelensis* infective third stage larvae (L3) at various time points (Table 4). Some extracts showed nematicide activity in a time-dependent manner since, by increasing the incubation time, the IC_50_ values decreased. The most active extracts against *S. venezuelensis* at 72 h of treatment were *R. chalepensis*, *J. spicigera*, *J. dioica*, *M. tenuiflora*, and *S. mexicanum*. Only *A. adstringens* did not affect *S. venezuelensis* viability (LC_50_ > 1000 µg/mL). Furthermore, all extracts were more toxic to *S*. *venezuelensis* than to mammalian cells. However, the only extracts that presented a SI > 1 were those of *R. chalepensis*, *M. tenuiflora*, *H. inuloides*, *S. mexicanum*, *P. decompositum*, *J. dioica,* and *S. aspera* (Table 4).

Since *R. chalepensis* extract showed the highest SI (SI = 2.58) against *T. spiralis*, we evaluated its partitions obtained by continuous Soxhlet extractions against *S. venezuelensis*. The *n*-hexane partition showed the highest activity, followed by the chloroform partition and the methanol partition at any time point post-treatment (Table 5). Furthermore, the *n*-hexane partition presented the highest SI, followed by the methanol and chloroform partitions (Table 5).

### 2.4. Identified Compounds in Ruta chalepensis n-Hexane Partition

**Figure 1:**
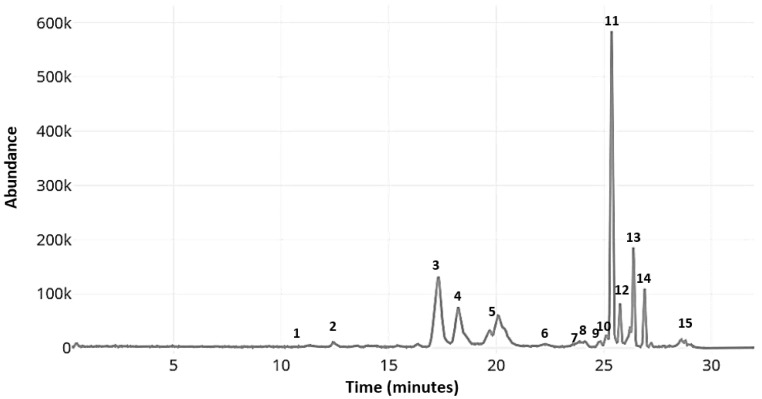
LC-ESI-MS/MS chromatogram obtained from the *n*-hexane partition of *R. chalepensis*.

Analysis of *R. chalepensis n*-hexane partition using LC-MS/MS revealed the presence of 15 compounds (Table 6, Supplementary Material File S1). Most of them corresponded to the chemical class of coumarins and derivatives (8/15 = 53%), followed by the indoles and derivatives (2/15 = 13%), quinolines and derivatives (2/15 = 13%), benzofurans (1/15 = 7%), flavonoids (1/15 = 7%), and fatty acyls (1/15 = 7%). The LC-MS/MS chromatogram of these compounds is shown in Figure 1.

**Figure 2:**
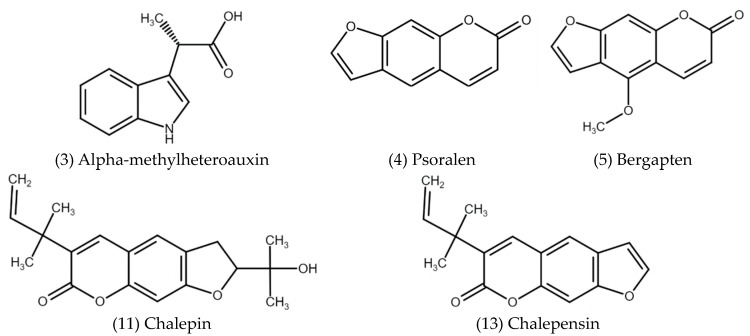
Structure of major compounds identified in the *n*-hexane partition of *R. chalepensis*.

The most abundant compounds present in the *n*-hexane partition were chalepin, psoralen, alpha-methylheteroauxin, bergapten, and chalepensin (Figure 2). The remaining 10 identified compounds showed an abundance of less than 4.8% (Table 6).

## 3. Discussion

*T. spiralis* and *S. stercolaris* are two parasites of medical importance that each year present high incidence rates worldwide, particularly in tropical and subtropical countries [3,7]. Nevertheless, the lack of suitable nematocidal drugs [14] and the emergence of drug-resistant nematodes in veterinary contexts [24] prompt the search for new treatments. For this reason, this study aims to screen extracts of Mexican medicinal plants as potential sources of novel nematocidal compounds. A broad range of plants was evaluated to identify those with greater antiparasitic potential, enabling the subsequent implementation of a targeted bioassay to discover the active compounds.

In our study, only 6 out of 12 evaluated plants had activity against *T. spiralis*, with *R. chalepensis*, *J. spicigera*, and *J. dioica* being the most active plants. Additionally, 11 plants had activity against *S. venezuelensis*, with *R. chalepensis* and *M. tenuiflora* being the most active plants. We can find in the literature multiple reports on the antiparasitic effect of *R. chalepensis* against various species, including *Meloidogyne incognita*, *M. javanica* [25], *Teladorsagia circumcinta*, *Haemonchus contortus*, and *Trichostrongylus* spp. [26]. In addition, *J. spicigera* has been shown to have activity against the trematodes *Fasciola hepatica* [27] and *Schistosoma mansoni* [28], and *M. tenuiflora* had activity against *Trypanosoma cruzi*, *Trichomonas vaginalis* [29], and *H. contortus* [30]. Despite *J. dioica* not being reported to possess antiparasitic activity, one of its compounds, naphthoquinone 4a, was active against *Taenia crassiceps* [31]. This emphasizes the antiparasitic potential of these plants.

Moreover, countless scientific publications have reported the activity of many plant extracts and isolated compounds, without determining SIs limiting their significance [32]. To assess the toxicity of plant extracts and calculate SIs in this study, we used Vero cells, which are commonly employed as a standard model for conducting cytotoxicity assessments of plant extracts and other chemical components relevant to studies exploring their anthelmintic properties [33]. For *T. spiralis*, we obtained SIs from 1.18 to 22.40, and in *S. venezuelensis*, the SIs were from 0.05 to 3.77. However, it is reported that for parasites, a crude extract may be assumed bioactive and non-toxic if SI > 1, since this increases the likelihood that its toxic and parasitic components are different [34], indicating that most of the evaluated plants in this study presented acceptable antiparasitic activity. Furthermore, in a previous study published by our working group, we previously evaluated the hemolytic, anti-hemolytic, and cytotoxic activity of the crude methanolic extracts of the plants used in this investigation against human peripheral blood mononuclear cells, finding that the extracts did not have hemolytic or cytotoxic effect on these cells [21], which exhibits their low toxicity. This opens the possibility of using these plant extracts as alternative treatments against trichinellosis and strongyloidiasis, as well as other parasitic diseases.

Since *R. chalepensis* extract showed the highest activity against both species of parasites, its partitions were obtained by continuous Soxhlet extractions to evaluate their activity. In both parasites, the *n*-hexane partition possessed the highest activity, followed by methanol and chloroform partitions. As the *n*-hexane partition showed the highest antiparasitic activity, we selected it to identify its main compounds due to their relevance. Using LC-MS/MS, we identified 15 compounds. Some of them have been previously reported as major compounds in *R. chalepensis,* such as chalepin, chalepensin, rutamarin [35], psoralen, bergapten [36], and dictamnine [37]. Other molecules have been identified in plants of the Rutaceae family, such as rutacultin [38], loliolide [39], and N-methylflindersine [40]. Additionally, other compounds have been identified in other plants as 13-oxo-ODE [41], bisgerayafoline A [42], 3-(1,1-dimethylallyl)-8-hydroxy-7-methoxycoumarin [43], and osthenol [44]. Therefore, this is the first report of the identification of these compounds in *R. chalepensis*.

Plants produce a wide range of secondary metabolites that have various biological activities, including antiparasitic effects [17]. The anthelmintic activity of plant extracts is linked to the presence of biologically active compounds such as condensed tannins, flavonoids, coumarins, steroids, terpenoids, alkaloids, and saponins [17]. These secondary metabolites exert their effect on helminths in different ways, depending on the different stages of the parasite’s development. These compounds may cause damage to the cuticle, interfering with motility or feeding, thus harming the growth and reproduction of helminths [45]. Most of the identified compounds in the *R. chalepensis n*-hexane partition belong to the chemical class of coumarins and derivatives (53%), which have been reported as strong antiparasitic agents against various nematodes [46]. Therefore, this chemical group may be responsible for the nematocidal activity of *R. chalepensis*. In addition, the most abundant compound identified was chalepin (29.97%), whose antiparasitic activity against *Entamoeba histolytica* [47] and *T. cruzi* [48] has been previously reported, suggesting its possible antiparasitic activity against *T. spiralis* and *S. venezuelensis*. Likewise, this compound has demonstrated various biological properties, such as antimicrobial activity against clinically significant bacteria [35] and cytotoxicity against cancer cell lines [49].

However, it is necessary to continue with the biotargeted fractionation to isolate and identify the bioactive compounds with antiparasitic activity. Additionally, it would be essential to assess the compounds in an in vivo murine model to confirm their antiparasitic activity and exclude any potential toxicity.

## 4. Materials and Methods

### 4.1. Plant Material and Extracts Preparation

The taxonomic identification of the plants used in this study was previously reported in a previous publication [21] and is shown in Table 1. Taxonomic validation of plant names and families was performed using ThePlantList website http://www.theplantlist.org (accessed 22 September 2023).

Plant extracts were obtained through Soxhlet extraction, following the methodology previously reported [21]. In this process, 25 g of each dried and milled plant sample was introduced into a Soxhlet extractor containing 0.5 L of absolute methanol (CTR Scientific, Monterrey, Nuevo León, Mexico). Due to methanol’s high polarity and its ability to solvate low-polarity compounds, it enables the isolation of a wide range of secondary metabolite groups present in plants [50]. Extraction was conducted over a 48 h period, and the resulting extracts were filtered and concentrated using a rotary evaporator (Buchi R-3000; Brinkman Instruments, Inc., Westbury, NY, USA) under reduced pressure at 40 °C in a water bath. The extraction yield for each extract was determined using the formula (1) provided below. Subsequently, 50 mg of each extract were dissolved in 1 mL of dimethyl sulfoxide (DMSO; Sigma-Aldrich, St. Louis, MO, USA). The solutions were sterilized by filtration using 0.22 µm pore size membrane filters (Corning Incorporated, Corning, NY, USA) and stored at −20 °C until needed. For the assays, serial working dilutions were prepared from the stock solutions using Luria–Bertani broth for *T. spiralis*, distilled water for *S. venezuelensis*, and DMEM medium for Vero cells. In each assay, 100 µL of each dilution was combined with 100 µL of the parasite or cell suspension, resulting in a final volume of 200 µL per well. It is noteworthy that the final concentration of DMSO in all culture conditions was maintained below 1% (*v/v*) to ensure no adverse effects on parasite and cell viability.

(1) $$\%\;Yield=\frac{Final\;weight\;of\;dry\;extract}{Initial\;weight\;of\;the\;plant}\times 100$$

### 4.2. Ethical Statement

Animal procedures complied with Spanish (RD 53/2013) and European Union (European Directive, 2010/63/CE) guidelines regarding animal experimentation for the protection and humane use of laboratory animals. In the accredited Animal Experimentation Facilities (registration number: PAE/SA/001) of the University of Salamanca (USAL), all procedures were conducted. The USAL’s Ethics Committee approved the procedures used in this study (CBE 335 CEI 1080). All efforts were made to minimize animal suffering. Animals were housed in standard polycarbonate cages under controlled 12 h light and dark cycles, with a temperature between 23 °C and 25 °C, and food and water ad libitum.

### 4.3. In Vitro Nematocidal Activity of Plant Extracts Against Trichinella spiralis

The infective first-stage larvae (L1) of *T. spiralis* were initially isolated in 1962 from a naturally infected wildcat (*Felis silvestris*) in Pola de Lena (Asturias, Spain) [51]. The cycle of *T. spiralis* was maintained by serial passage in 4-week-old male CD1 mice weighing 25 g to 30 g. Mice were orally infected with 600 L1 suspended in 500 µL of phosphate-buffered saline (PBS, pH 7.4). Mice were euthanized 40 days post-infection by cervical dislocation under anesthesia, and larvae encysted in animal muscles were obtained by enzymatic digestion. For this, a necropsy was first performed to obtain the carcass, without skin and viscera, to be weighed and ground. The resulting biological material was suspended in sterile 0.85% saline solution (100 mL/10 g of carcass), pepsin (0.5 g/10 g of carcass; Sigma-Aldrich), and 35% hydrochloric acid (0.7 mL/10 g of meat; Panreac, Barcelona, Spain). The mixture was then incubated for 90 min at 37 °C and 200 rpm in an incubator shaker (Optic Ivymen System, Barcelona, Spain) and filtered through sterile gauze in a settling cup [52]. Larvae were washed five times with sterile 0.85% saline solution, and viability and concentration were determined using a light microscope prior to the experiments.

We placed 35 to 50 *T. spiralis* larvae per well in flat-bottomed 96-well microplates (Corning Incorporated, Corning, NY, USA) in 100 mL of Luria–Bertani (LB) broth (Sigma-Aldrich), plus 1% penicillin/streptomycin solution (Gibco, Grand Island, NY, USA). The larvae were treated with working solutions ranging from 3.9 µg/mL to 1000 µg/mL. Albendazole (Sigma-Aldrich) and mebendazole (Sigma-Aldrich) at 20 µM were used as positive controls, while LB broth with or without 1% DMSO was the negative control. Larvae were incubated (RS Biotech Galaxy S incubator; Aberdeen, UK) for 72 h at 37 °C in a humidified atmosphere [51]. Larvae mortality and morphology were evaluated in photographs at 72 h under light microscopy at 40× magnification.

### 4.4. In Vitro Nematocidal Activity of Extracts Against Strongyloides venezuelensis

The *S. venezuelensis* life cycle was maintained by successive passages in 4-week-old male Wistar rats weighing 150 g to 200 g to complete their life cycle. Rats were subcutaneously injected with 12,000 infective third-stage larvae (L3) in 500 µL of PBS. Rat feces were collected from 5 to 18 days post-infection and cultured in 250 mL polyethylene containers with vermiculite and distilled water for 4 to 7 days at 28 °C in a humid atmosphere (SANYO Electric Co., Ltd., Fujioka, Japan). L3 were harvested by the Baermann method. They were washed three times with distilled water, and viability was determined by light microscopy prior to experiments [53,54].

We placed 100 to 150 larvae per well of *S. venezuelensis* in flat-bottomed 96-well microplates (Corning Incorporated) in 100 mL of distilled water and incubated at 28 °C for 30 min to allow adaptation. Subsequently, they were treated with extracts at concentrations ranging from 3.9 µg/mL to 1000 µg/mL. We used 10 µM ivermectin (Sigma-Aldrich) as a positive control and distilled water with or without 1% DMSO as negative controls. L3 were incubated for 72 h at 28 °C in a humid atmosphere (SANYO Electric Co., Ltd.), and mortality was evaluated at 24 h, 48 h, and 72 h based on the absence of mobility under light microscopy when excited by visible light. Mortality was determined as the lack of movement detected for at least one minute of observation under the microscope at 40× magnification [55].

### 4.5. Cytotoxic Activity of Plant Extracts Against Vero Cells

African green monkey kidney epithelial cells (Vero; ATCC CCL-81) were cultured in Dulbecco’s modified Eagle medium (DMEM; Gibco, Grand Island, NY, USA) supplemented with 10% heat-inactivated fetal bovine serum (FBS; Gibco), 2 g/L sodium bicarbonate (NaHCO_3_), and 1% penicillin/streptomycin solution (Gibco) (referred to as complete DMEM medium). Cells were cultured at 37 °C in an atmosphere of 5% CO_2_ [56]. Vero cells were seeded at a concentration of 1 × 10^4^ cells per well in flat-bottomed 96-well microplates (Corning Incorporated) in complete DMEM medium. After 24 h of incubation at 37 °C in an atmosphere of 5% CO_2_ in air, cells were treated with methanol extracts at concentrations ranging from 3.9 µg/mL to 1000 µg/mL, with culture medium with or without 1% of DMSO as negative controls. Following 48 h of incubation, cell viability was determined using the colorimetric 3-[4,5-dimethylthiazol-2-yl]-2,5-diphenyltetrazoliumbromide (MTT; Affymetrix, Cleveland, OH, USA) reduction assay. MTT was added at a final concentration of 0.5 mg/mL to each well and incubated at 37 °C for an additional 3 h. Plates were then decanted, and formazan crystals were dissolved in 100 µL of DMSO. Optical densities (OD) were determined at 570 nm using a MULTISKAN GO microplate reader (Thermo Fisher Scientific, Waltham, MA, USA) [57]. Percentage growth inhibition was calculated using the following formula (2):

(2) $$\%\;Cell\;growth\;inhibition=100-\left(\frac{{OD}_{570}\;Treated\;cells}{{OD}_{570}\;Untreated\;cells}\times 100\right)$$

### 4.6. Determination of Selectivity Index of Plant Extracts

In this study, Vero cells were used as a mammalian cell model for testing unspecific cytotoxicity of the extracts. Selectivity index (SI) was determined to assess parasitic activity relative to toxicity in normal mammalian cells, where high SI indicates potent activity with low cell toxicity [58]. An SI value greater than 1 for a crude extract increases the likelihood that its toxic and nematocidal components are different [34]. Plant extract SIs were calculated by dividing the half maximal inhibitory concentration (IC_50_) in normal mammalian cells (Vero) by the mean lethal concentration (LC_50_) in parasites (*T. spiralis* or *S. venezuelensis*), using the following formula (3):

(3) $$SI=\frac{{IC}_{50}\;mammalian\;cells}{{LC}_{50}\;parasite}$$

### 4.7. Ruta chalepensis Extract Partition Preparation

We selected the *R. chalepensis* crude methanolic extract to obtain its partitions since it showed the best in vitro nematocidal activity based on its SIs. Partitions were obtained by continuous Soxhlet extractions using solvents of increasing polarity. For this process, 5 g of the crude methanolic extract of *R. chalepensis*, filtered and concentrated to dryness, was subjected to sequential extraction with *n*-hexane, chloroform, and finally methanol, using 250 mL of solvent for each step. Each extraction lasted 48 h, and the residual methanolic extract was carried forward to the next solvent stage. The partitions obtained were filtered and concentrated as previously described for the crude methanolic extract [59]. Nematocidal activity against *T. spiralis* and *S. venezuelensis* was evaluated as detailed above.

### 4.8. Liquid Chromatography–Tandem Mass Spectrometry Analysis of Ruta chalepensis n-Hexane Partition

We selected the *n*-hexane partition of *R. chalepensis* for compound identification because it exhibited the highest nematocidal activity, as indicated by its SIs against both parasites. Therefore, the compounds in this partition are those with the greatest antiparasitic potential compared to the other extracts. For analysis, *R. chalepensis n*-hexane partition suspended in water-acetonitrile (80:20) was injected (2 µL or 600 ng) into an Agilent 1260 Infinity LC (Agilent Technologies, Inc., Santa Clara, CA, USA). The LC-MS/MS analysis followed the protocol previously described [60]. Nanospray ionization in positive mode was used for MS data acquisition under the following conditions: capillary voltage (1850 V), gas temperature (350 °C), drying gas flow (5 L/min), skimmer voltage (65 V), octapole RF (750 V), fragmentor voltage (175 V), and a spectra acquisition rate (4 spectra/s) over a mass range of 110–2000 m/z. For MS2 data, a narrow isolation window (1.3 m/z), a spectra acquisition rate (3 spectra/s), and a maximum of 5 precursors per cycle over 50–2000 m/z were used. Active exclusion was enabled for 2 spectra with a release time of 0.25 min. Collision energy was ramped with slope and offset values of 6 and 4.

The LC-MS/MS datasets were analyzed using open-access software packages and online platforms. We used the global natural products social molecular networking web platform (GNPS, https://gnps.ucsd.edu, accessed on 5 March 2023) for automated structural analysis. Additionally, as a complementary tool, we employed the CSI:FingerID package from SIRIUS software version 4.9.12 (https://bio.informatik.uni-jena.de/software/sirius/, accessed on 5 March 2023). The chemical classes of the identified metabolites were automatically determined using the Classyfire web-based application (http://classyfire.wishartlab.com, accessed on 10 March 2023) [60].

### 4.9. Statistical Analysis

All statistical analyses were carried out using the GraphPad Prism 8 statistical package (GraphPad Software Inc., San Diego, CA, USA). The data presented herein represents the means ± standard deviations (SD) of triplicates derived from a minimum of three independent experiments, with a confidence level of 95%. To assess the significant differences among the tested extracts, a one-way analysis of variance (1-way ANOVA) was conducted. Post hoc analysis used Tukey’s test to determine variations between treatment means. The Probit test was employed for the computation of IC_50_ and LC_50_ values.

## 5. Conclusions

This study demonstrated the antiparasitic effect of some plant’s crude methanol extracts against *T. spiralis* and *S. venezuelensis* in a time- and concentration-dependent manner. Most of the evaluated extracts showed potential nematocidal activity and selective toxicity against parasites, as compared with mammalian cells (SI > 2), suggesting the possibility of using these extracts as alternative treatments for these parasites. In particular, *R. chalepensis* crude methanol extract showed significant nematocidal activity against both parasites, and its *n*-hexane partition was the most active. It also highlights the chance to isolate and identify new compounds with antiparasitic activity from these plants, especially focusing on the *n*-hexane partition of *R. chalepensis*.

## Figures and Tables

**Table 1:** Identification of plants.

Scientific Name	Mexican Common Name	Family	Plant Section	Yield (%)	Voucher Id	Traditional Use [23]
*Amphipterygium adstringens* (Schltdl.) Standl.	Cuachalalate	Anacardiaceae	B	24.82	30642	Gastrointestinal disorders, respiratory conditions, cancer, skin lesions, and parasitic infections.
*Artemisia ludoviciana* Nutt.	Estafiate	Compositae	SLF	17.31	30643	Gastrointestinal disorders, respiratory conditions, and intestinal parasitic infections.
*Cymbopogon citratus* (DC.) Stapf.	Zacate limón	Poaceae	LS	23.04	30644	Gastrointestinal disorders, fever, and headaches.
*Heterotheca inuloides* Cass.	Árnica	Compositae	F	21.21	30646	Healing, disinfectant, anti-inflammatory, analgesic, and gastrointestinal disorders.
*Jatropha dioica* Sessé	Sangre de drago	Euphorbiaceae	R	19.58	30648	Hair loss, irritated eyes, and skin infections.
*Justicia spicigera* Schltdl.	Muicle	Acanthaceae	SLF	27.37	30649	Gastrointestinal disorders, respiratory issues, and fever.
*Larrea tridentata* (Sessé and Moc. ex DC.) Coville	Gobernadora	Zygophyllaceae	LS	26.61	30650	Urinary issues, gastrointestinal disorders, and foot infections.
*Mimosa tenuiflora* (Willd.) Poir.	Tepezcohuite	Leguminosae	B	11.25	30651	Treat skin issues such as wounds and burns.
*Psacalium decompositum* (A. Gray) H. Rob. and Brettell	Matarique	Compositae	R	14.43	30652	Rheumatic conditions, diabetes, kidney pain, colds, and gastrointestinal disorders.
*Ruta chalepensis* L.	Ruda	Rutaceae	LS	19.40	30654	Gastrointestinal disorders, intestinal parasitic infections, fever, and ear infections.
*Semialarium mexicanum* (Miers) Mennega	Cancerina	Celastraceae	Bark	11.14	30647	Treat ulcers and wounds.
*Smilax aspera L.*	Zarzaparilla	Smilacaceae	R	18.26	30655	Gastrointestinal disorders, menstrual pain, syphilis, and cancer.

B: bark; SLF: stems, leaves, and flowers; LS: leaves and stems; F: flowers; R: root.

**Table 2:** In vitro nematocidal activity against *T. spiralis* and cytotoxic activity against Vero cells of crude methanol plant extracts.

Plant	µg/mL		SI
	LC_50_ *T. spiralis*	IC_50_ Vero	
*Amphipterygium adstringens*	>1000 ^†^	>1000 ^†^	ND
*Artemisia ludoviciana*	>1000 ^†^	295.5 ± 6.6 ^c^	ND
*Cymbopogon citratus*	>1000 ^†^	31.7 ± 3.9 ^a^	ND
*Heterotheca inuloides*	>1000 ^†^	789.1 ± 5.7 ^h^	ND
*Jatropha dioica*	134.8 ± 7.6 ^c^	404.1 ± 5.6 ^d^	2.30
*Justicia spicigera*	44.8 ± 6.3 ^b^	168.9 ± 3.5 ^b^	3.77
*Larrea tridentata*	450.6 ± 9.2 ^e^	531.9 ± 7.2 ^e^	1.18
*Mimosa tenuiflora*	>1000 ^†^	>1000 ^†^	ND
*Psacalium decompositum*	148.5 ± 8.6 ^c^	911.9 ± 7.3 ^j^	6.14
*Ruta chalepensis*	28.2 ± 3.7 ^a^	631.8 ± 6.4 ^f^	22.40
*Semialarium mexicanum*	>1000 ^†^	678.7 ± 2.8 ^g^	ND
*Smilax aspera*	188.8 ± 3.5 ^d^	893.4 ± 6.4 ^i^	4.73
Albendazole	0.0018 ± 0.0001	NE	-
Mebendazole	0.0033 ± 0.0005	NE	-

Data are mean ± SD of the LC_50_ or IC_50_ (µg/mL) for each extract against *T. spiralis* and Vero cells. Different letters within the same column are significantly different, analyzed by the post hoc Tukey test (*p* < 0.05). SI represents IC_50_ of Vero cells divided by LC_50_ of *T. spiralis* at 72 h. ^†^ As LC_50_ or IC_50_ was above 1000 µg/mL, these values were not considered for Tukey analysis. NE: Not evaluated. ND: Not determined because this extract did not show activity at any of the evaluated concentrations.

**Table 3:** *R. chalepensis* partitions yields and in vitro nematocidal activity against *T. spiralis* activity and cytotoxicity to Vero cells.

Partition	Yield %	µg/mL		SI
		LC_50_ *T. spiralis*	IC_50_ Vero	
*n*-Hexane	1.55	19.0 ± 3.9 ^a^	147.6 ± 4.4 ^b^	7.77
Chloroform	0.90	29.3 ± 5.9 ^b^	65.4 ± 3.8 ^a^	2.23
Methanol	7.76	161.5 ± 4.4 ^c^	994.7 ± 6.7 ^c^	6.16

Data are mean ± SD of the LC_50_ or IC_50_ (µg/mL) for each *R. chalepensis* partition against *T. spiralis* and Vero cells. Different letters within the same column are significantly different by the post hoc Tukey test (*p* < 0.05). SI represents IC_50_ of Vero cells divided by LC_50_ of *T. spiralis* at 72 h.

**Table 4:** In vitro nematocidal activity of crude methanol plant extracts against *S. venezuelensis*.

Plant	LC_50_ in µg/mL			SI
	24 h	48 h	72 h	
*Amphipterygium adstringens*	>1000 ^†^	>1000 ^†^	>1000 ^†^	ND
*Artemisia ludoviciana*	>1000 ^†^	574.7 ± 4.7 ^e^	543.6 ± 5.1 ^f^	0.54
*Cymbopogon citratus*	945.0 ± 6.7 ^i^	622.5 ± 5.4 ^f^	586.1 ± 4.5 ^g^	0.05
*Heterotheca inuloides*	>1000 ^†^	588.7 ± 4.6 ^e^	556.9 ± 4.8 ^f^	1.42
*Jatropha dioica*	425.3 ± 4.9 ^b^	404.8 ± 4.3 ^c^	382.4 ± 4.6 ^c^	1.06
*Justicia spicigera*	319.4 ± 4.8 ^a^	294.6 ± 4.1 ^a^	271.4 ± 4.9 ^b^	0.62
*Larrea tridentata*	794.2 ± 5.3 ^f^	690.4 ± 4.4 ^g^	622.9 ± 4.5 ^h^	0.85
*Mimosa tenuiflora*	651.1 ± 4.7 ^d^	539.3 ± 6.7 ^d^	404.3 ± 5.9 ^d^	>2.46
*Psacalium decompositum*	768.6 ± 5.3 ^e^	736.6 ± 5.8 ^h^	684.5 ± 5.2 ^i^	1.33
*Ruta chalepensis*	840.2 ± 7.0 ^g^	348.7 ± 6.4 ^b^	244.8 ± 5.8 ^a^	2.58
*Semialarium mexicanum*	547.4 ± 4.9 ^c^	529.4 ± 4.5 ^d^	499.9 ± 6.1 ^e^	1.36
*Smilax aspera*	915.9 ± 5.6 ^h^	890.5 ± 4.8 ^i^	865.3 ± 4.5 ^j^	1.03
Ivermectin	1.8 ± 0.9	1.4 ± 0.3	1.3 ± 0.3	-

Data are mean ± SD of the LC_50_ (µg/mL) for each extract against *S. venezuelensis* at various times. Different letters within the same column are significantly different by the post hoc Tukey test (*p <* 0.05). SI represents the IC_50_ of Vero cells (shown in Table 2) divided by the LC_50_ of *S. venezuelensis* at 72 h. ^†^ As LC_50_ was above 1000 µg/mL, these values were not considered for Tukey analysis. ND: Not determined because this extract did not show activity at any of the evaluated concentrations.

**Table 5:** In vitro nematocidal activity of *Ruta chalepensis* partitions against *Strongyloides venezuelensis* at 24, 48, and 72 h.

Partition	LC_50_ in µg/mL			SI
	24 h	48 h	72 h	
*n*-Hexane	43.0 ± 3.9 ^a^	40.9 ± 3.5 ^a^	39.2 ± 3.1 ^a^	3.77
Chloroform	126.7 ± 4.7 ^b^	115.3 ± 5.7 ^b^	110.2 ± 6.4 ^b^	0.59
Methanol	367.1 ± 5.9 ^c^	339.1 ± 6.3 ^c^	314.7 ± 5.5 ^c^	3.16

Data are mean ± SD of the LC_50_ (µg/mL) for each partition of *R. chalepensis* against *S. venezuelensis* at various times. Different letters within the same column are significantly different by the post hoc Tukey test (*p <* 0.05). SI represents the IC_50_ of Vero cells (shown in Table 3) divided by the LC_50_ of *S. venezuelensis* at 72 h.

**Table 6:** LC-MS/MS compounds in *Ruta chalepensis n*-hexane partition.

No.	Compound	Experimental Mass	Theoretical Mass	Adduct	Mass Error (ppm)	Formula	RT	Relative %	Chemical Class
1	Loliolide	197.1170	197.1172	[M + H]^+^	−0.73	C_11_H_16_O_3_	11.2	0.54	BNZ
2	Rutin	611.1610	611.1607	[M + H]^+^	0.68	C_27_H_30_O_16_	12.4	0.38	FLV
3	Alpha-methylheteroauxin	190.0860	190.0863	[M + H]^+^	−0.94	C_11_H_11_NO_2_	17.1	12.03	IND
4	Psoralen	187.0380	187.0317	[M + H]^+^	−4.78	C_11_H_6_O_3_	18.1	15.82	CUM
5	Bergapten	217.0490	217.0495	[M + H]^+^	−2.11	C_12_H_8_O_4_	19.6	11.47	CUM
6	Dictamnine	200.0700	200.0706	[M + H]^+^	−2.64	C_12_H_9_NO_2_	20.4	4.63	QIN
7	3-(1,1-dimethylallyl)-8-hydroxy-7-methoxycoumarin	261.1110	261.1121	[M + H]^+^	−4.06	C_15_H_16_O_4_	23.9	3.03	CUM
8	Osthenol	231.1010	231.1016	[M + H]^+^	−2.14	C_14_H_14_O_3_	23.9	2.10	CUM
9	N-Methylflindersine	242.1180	242.1176	[M + H]^+^	2.15	C_15_H_15_NO_2_	24.8	0.77	QIN
10	Rutacultin	275.1280	275.1278	[M + H]^+^	1.06	C_16_H_18_O_4_	25.6	4.21	CUM
11	Chalepin	315.1600	315.1591	[M + H]^+^	3.14	C_19_H_22_O_4_	25.7	29.97	CUM
12	13-Oxo-ODE	277.2160	277.2164	[M − H_2_O + H]^+^	−2.72	C1_8_H_30_O_3_	26.1	1.42	FTA
13	Chalepensin	255.1020	255.1016	[M + H]^+^	1.98	C_16_H_14_O_3_	26.4	8.89	CUM
14	Rutamarin	357.1720	357.1697	[M + H]^+^	6.79	C_21_H_24_O_5_	26.8	4.71	CUM
15	Bisgerayafoline A	815.4860	815.4776	[M + H]^+^	9.62	C_55_H_62_N_2_O_4_	28.8	0.02	IND

RT: retention time in minutes; BNZ: benzofurans; FLV: flavonoids; IND: indoles and derivatives; CUM: coumarins and derivatives; QIN: quinolines and derivatives; FTA: fatty acyls.

## Data Availability

The datasets generated or analyzed during the present study are available from the corresponding authors.

## Supplementary Materials

The following supporting information can be downloaded at: https://www.mdpi.com/article/10.3390/plants13243484/s1, File S1: The mass spectra (MS) of the compounds identified in the *n*-hexane fraction of *Ruta chalepensis*.
