# Supplementary material for: In Vitro Anthelmintic Effect of Mexican Plant Extracts and Partitions Against Trichinella spiralis and Strongyloides venezuelensis
Source: Plants (Basel). 2024 Dec 12;13(24):3484. doi: 10.3390/plants13243484 (PMC11728750; doi:10.3390/plants13243484)

plants

Article

In vitro Anthelmintic Effect of Mexican Plant Extracts and Fractions against *Trichinella spiralis* and *Strongyloides venezuelensis*

Nancy E. Rodríguez-Garza [1,2], Ricardo Gomez-Flores [1], Ramiro Quintanilla-Licea [3], Joel H. Elizondo-Luevano [2,3], César I. Romo-Sáenz [1,4], Miguel Marín [2], Javier Sánchez-Montejo [2], Antonio Muro [2], Rafel Peláez [2,*] and Julio López-Abán [2,*]

[1] Departamento de Microbiología e Inmunología, Facultad de Ciencias Biológicas, Universidad Autónoma de Nuevo León, San Nicolás de los Garza 66455, N.L., Mexico
[2] Grupo de Enfermedades Infecciosas y Tropicales (e-INTRO), Instituto de Investigación Biomédica de Salamanca – Centro de Investigación de Enfermedades Tropicales de la Universidad de Salamanca (IBSAL-CIETUS), Facultad de Farmacia, Universidad de Salamanca, 37007 Salamanca, Spain
[3] Departamento de Química, Facultad de Ciencias Biológicas, Universidad Autónoma de Nuevo León, San Nicolás de los Garza 66455, N.L., Mexico
[4] Facultad de Medicina y Ciencias Biomédicas, Universidad Autónoma de Chihuahua, 31109 Chihuahua, Mexico
* Correspondence: jlaban@usal.es (J.L.-A.); pelaez@usal.es (R.P.)

Supplementary Material

In this document, the mass spectra (MS) of the compounds identified in the *n*-hexane fraction of *Ruta chalepensis* are presented.

1. **Loliolide**

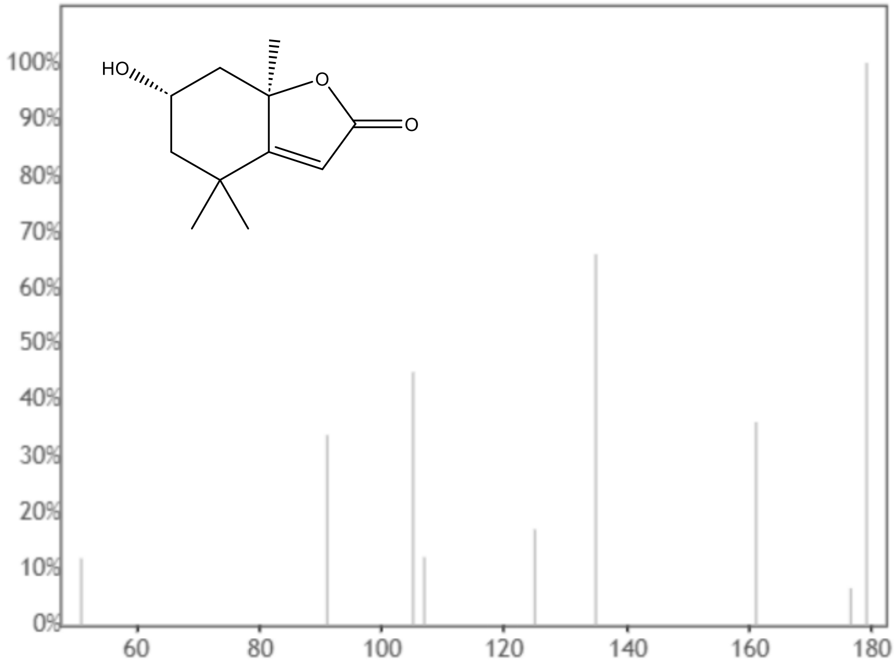

2. Rutin

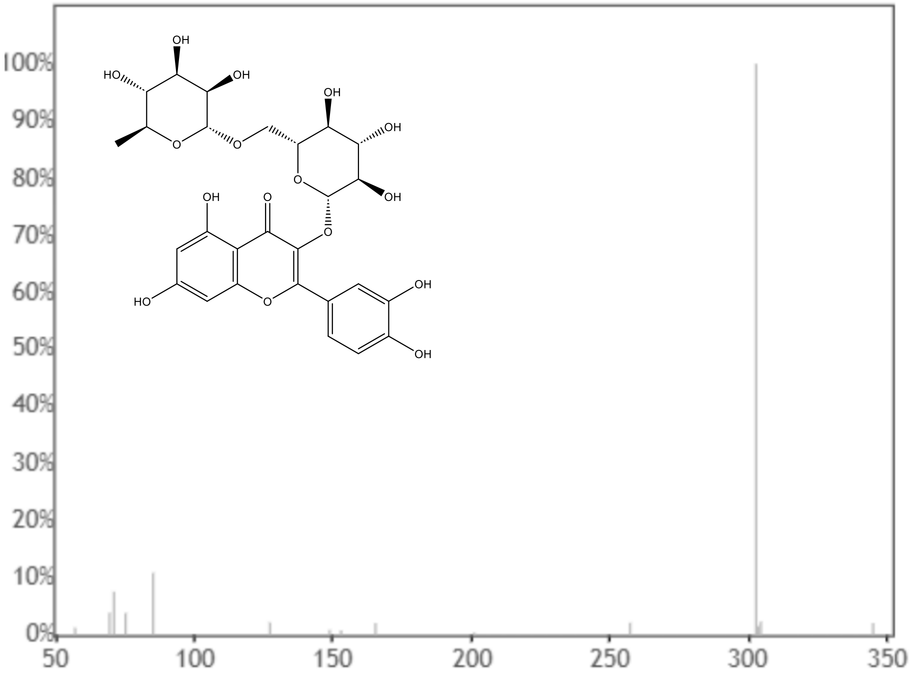

3. Alpha-methylheteroauxin

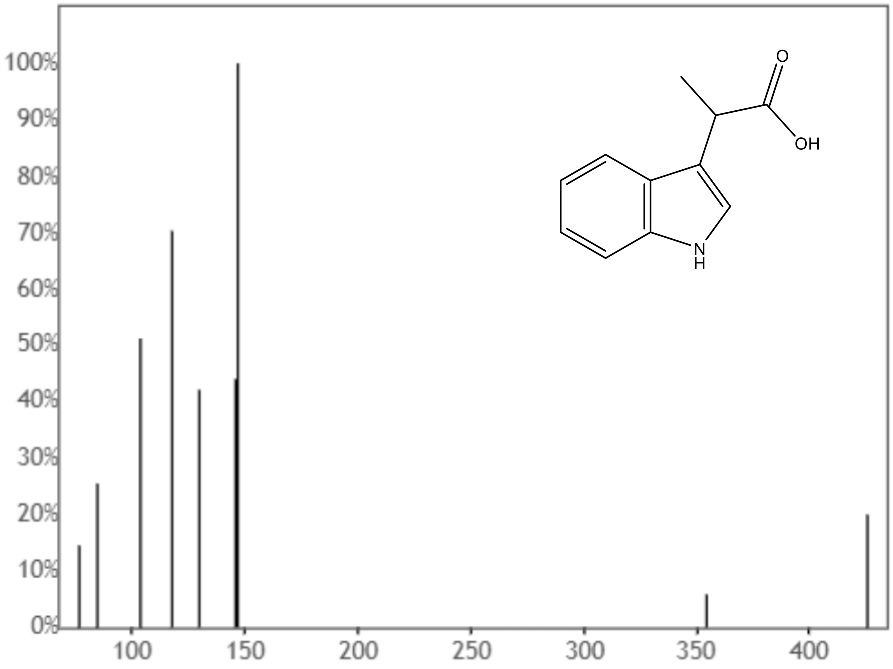

4. **Psoralen**

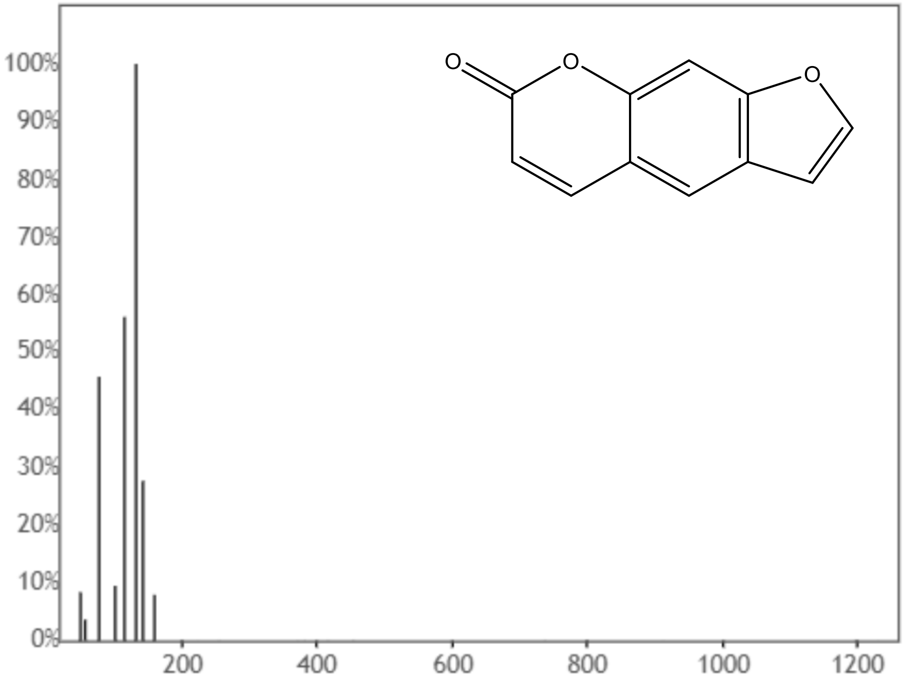

5. **Bergapten**

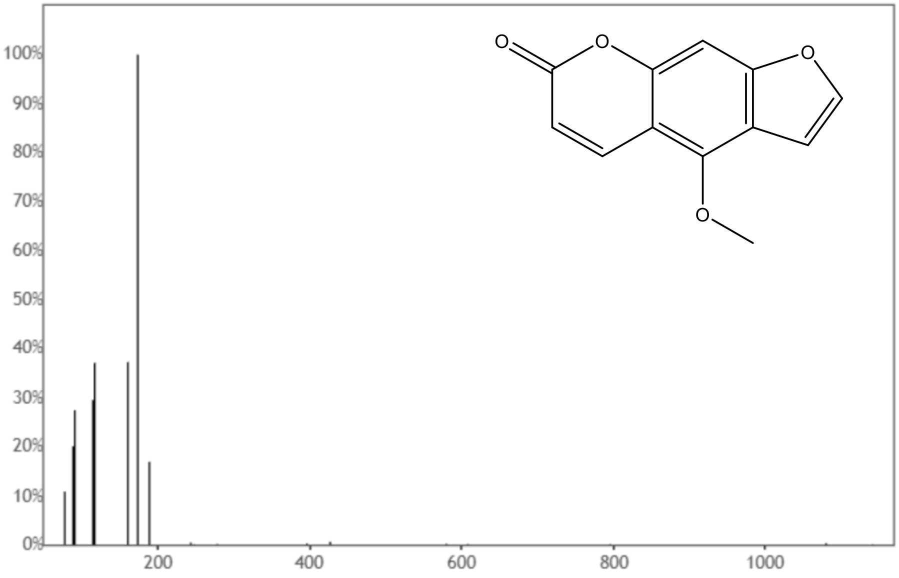

6. **Dictamnine**

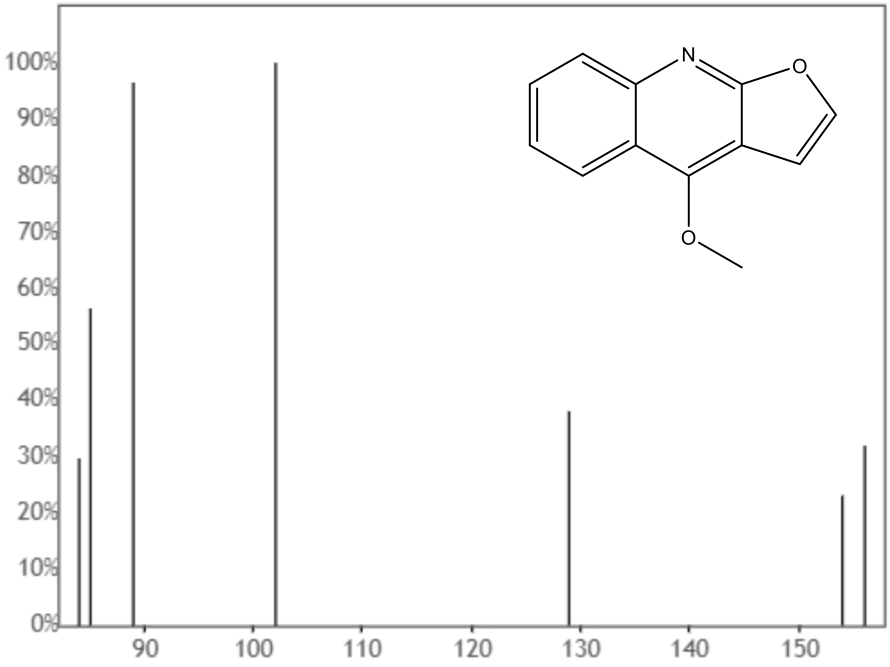

7. **3-(1,1-dimethylallyl)-8-hydroxy-7-methoxycoumarin**

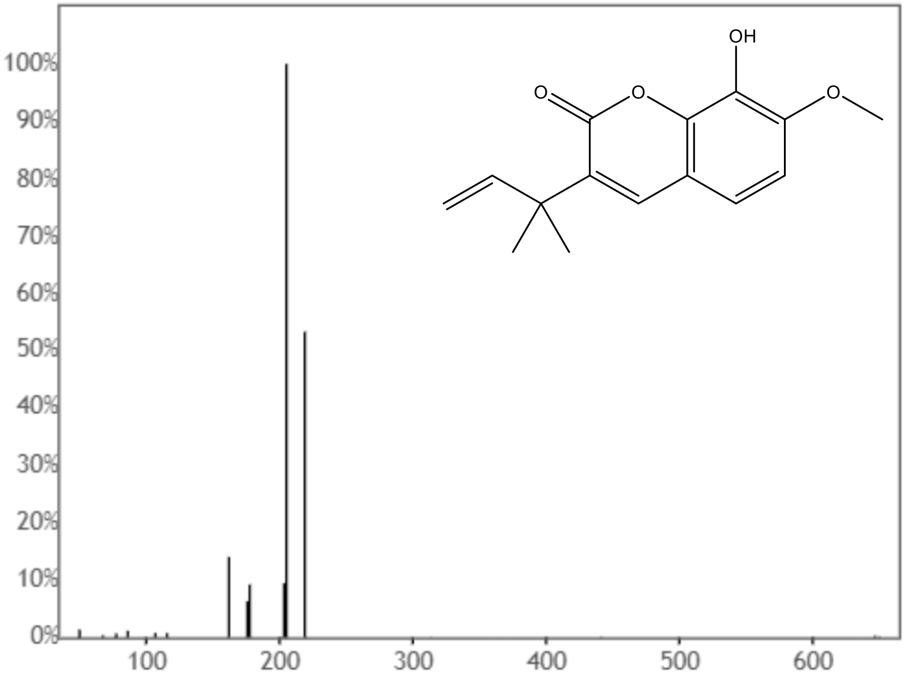

plants

MDPI

8. Osthenol

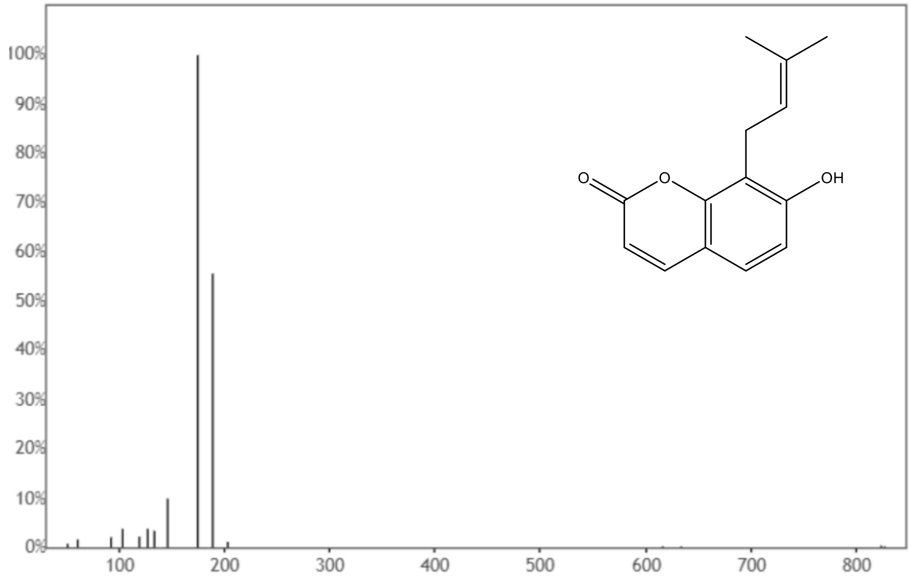

9. N-Methylflindersine

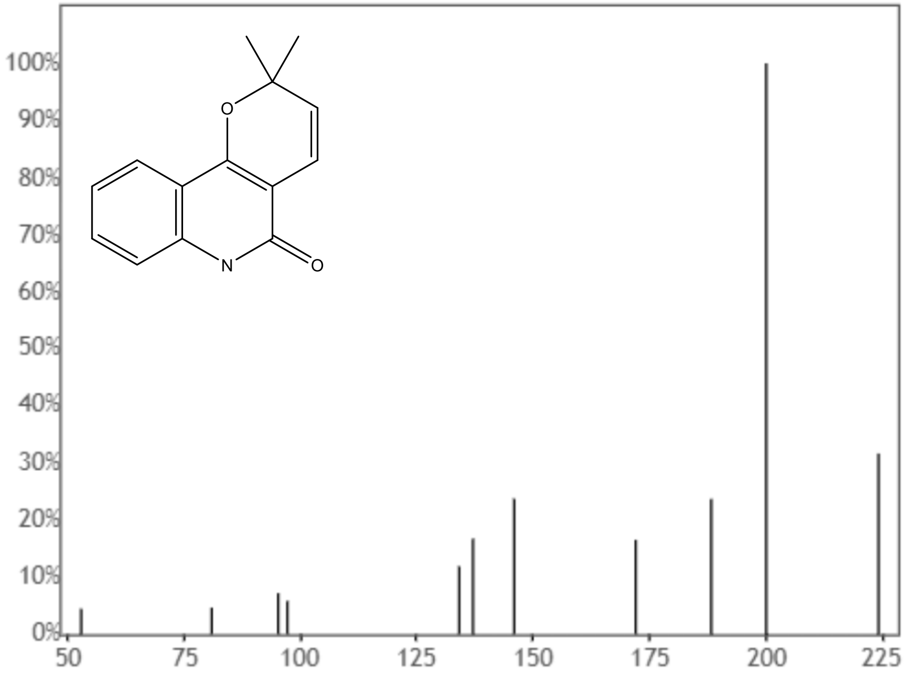

10. **Rutacultin**

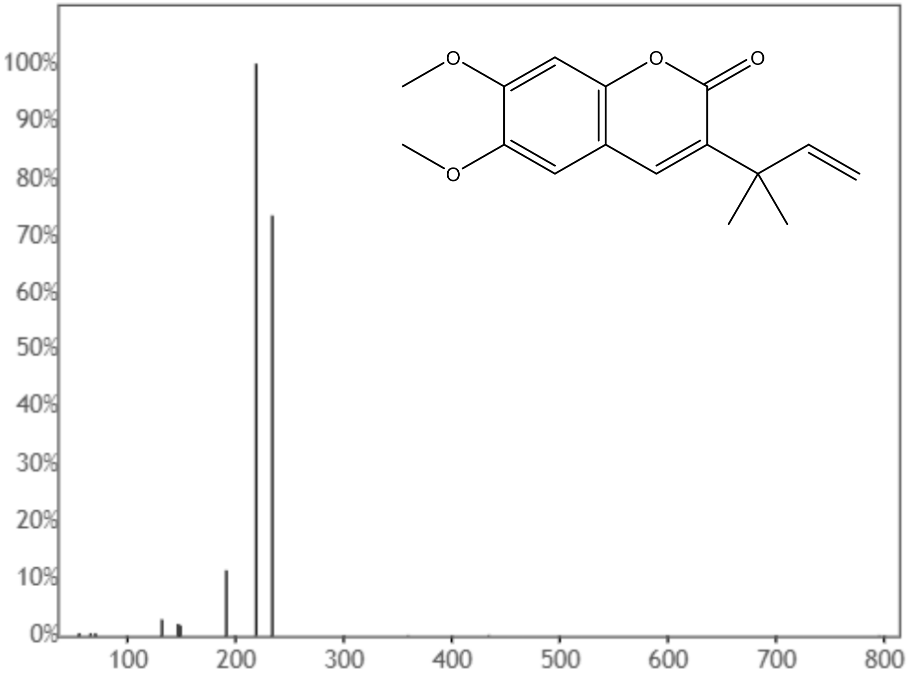

11. **Chalepin**

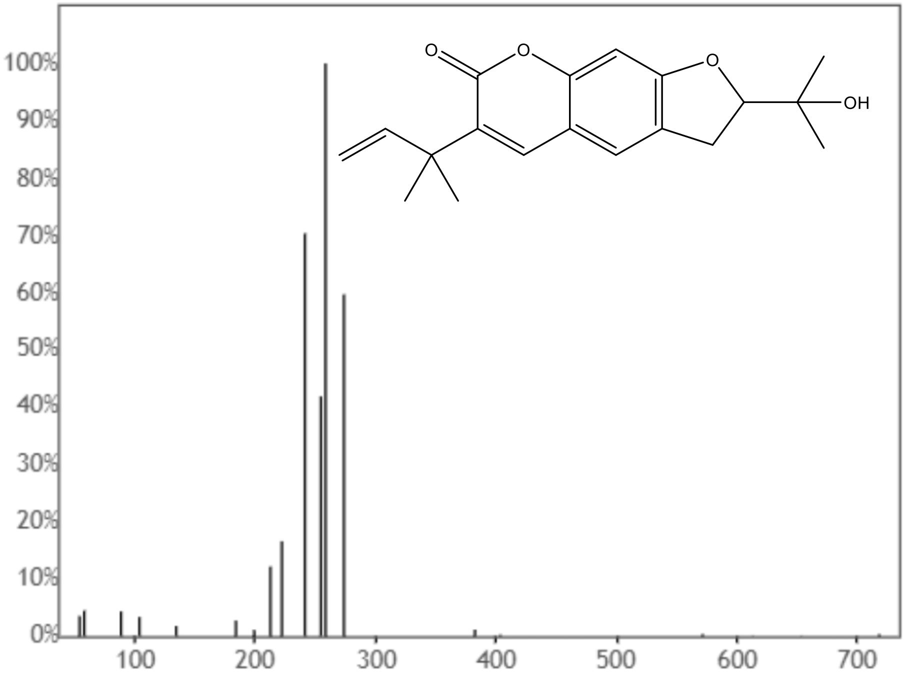

12. 13-Oxo-ODE

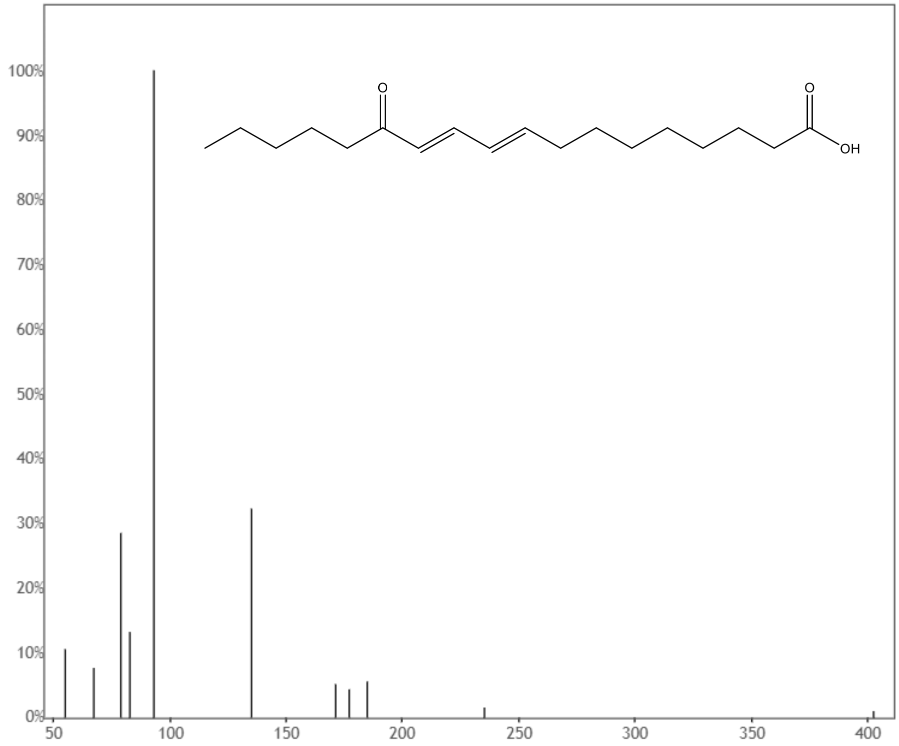

13. Chalepensin

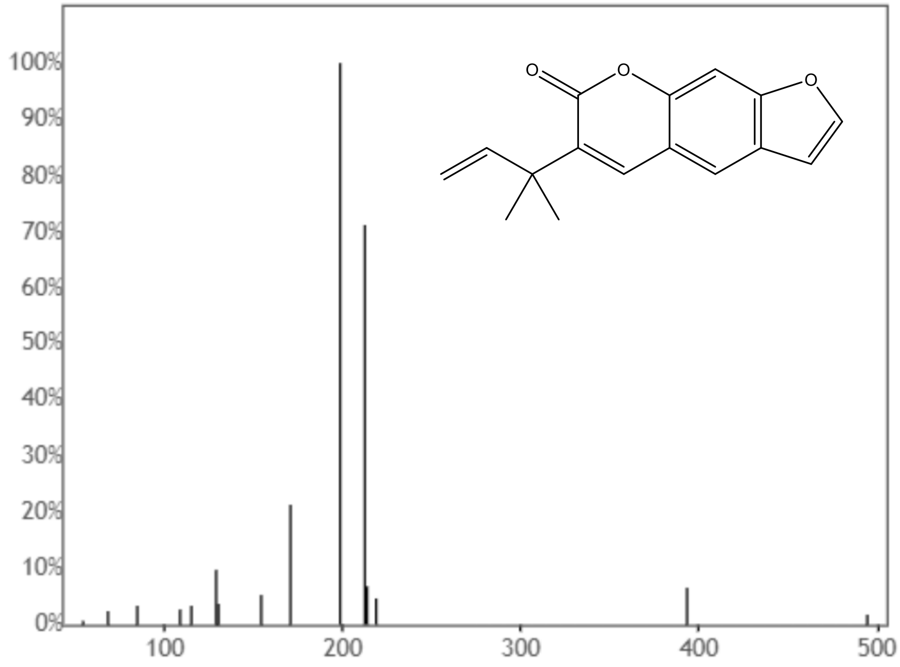

14. Rutamarin

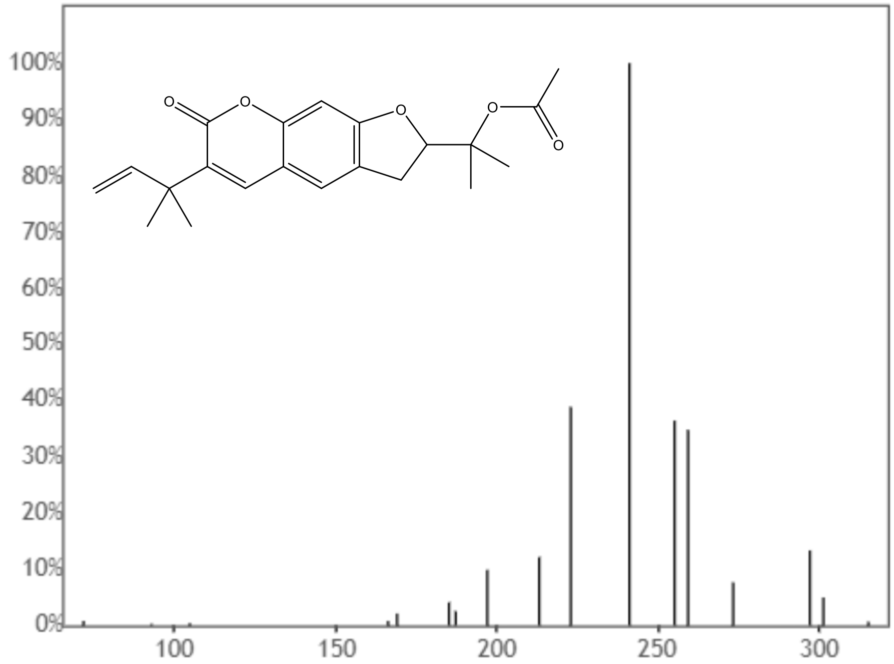

15. Bisgerayafoline A

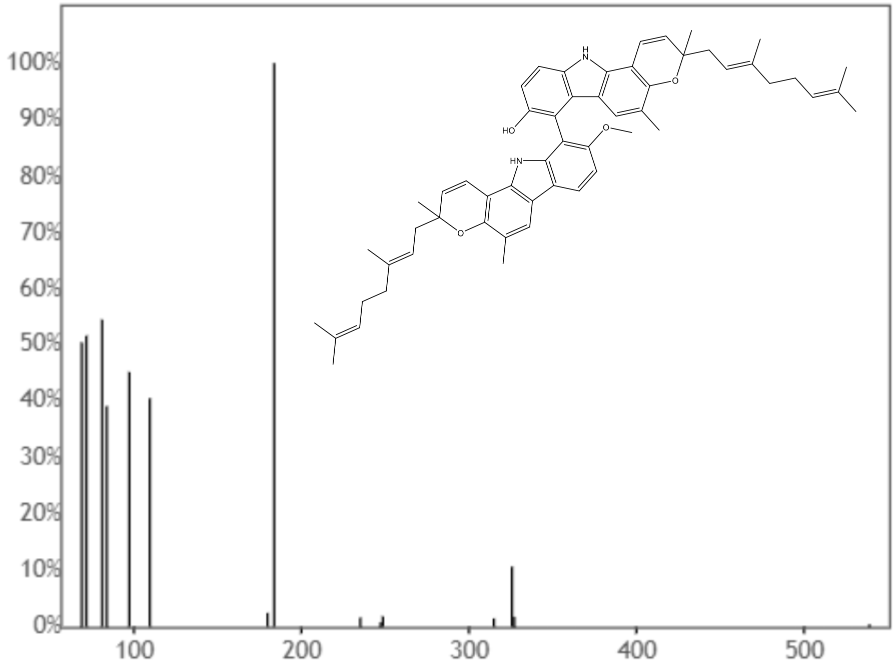

Supplement: Supplementary file 1 [file plants-13-03484-s001.zip › plants-3343580-supplementary.pdf]
